# From surviving to thriving: a process–ecological model of psychological resilience in doctoral students

**DOI:** 10.3389/fpsyg.2026.1767701

**Published:** 2026-03-05

**Authors:** Hang Zhao, Jin Qiang Ma, Cheng Zhang, Ming Lin Chen

**Affiliations:** 1 College of Life Science, Anhui Normal University, Wuhu, Anhui Province, China; 2 Anhui Business College, Wuhu, Anhui Province, China

**Keywords:** graduate education, grounded theory, process model, psychological development, psychological resilience

## Abstract

**Background:**

Doctoral students worldwide face considerable mental health challenges. Predominant research, often grounded in a pathological paradigm, has treated psychological resilience as a static trait, thereby overlooking its dynamic construction and ecological embeddedness within person–environment interactions.

**Aim:**

This study explores how doctoral students in high-pressure academic settings build psychological resilience through ongoing interaction with their ecosystems to transition from a state of survival to one of thriving.

**Methods:**

Using a constructivist grounded theory approach, we conducted semi-structured interviews with 24 doctoral students from Chinese universities. Data were analyzed through a process of constant comparative method and iterative coding.

**Results:**

The analysis yielded a process–ecological model of psychological resilience. This model identifies a four-stage, nonlinear developmental pathway: stress perception, cognitive restructuring, strategy integration, and value transcendence. This progression is powered by a dual-engine mechanism in which meaning-making provides direction and agency activation supplies motivation, all nested within and shaped by the dynamic interplay of individual, relational, and institutional ecosystems.

**Conclusion:**

The process-ecological model frames psychological resilience as a dynamic practice that co-evolves with academic identity formation. We advocate for a fundamental paradigm shift in the doctoral student support system—from individual-level remedial interventions toward the systematic cultivation of an enabling, ecological resilience system.

## 1 Introduction

Doctoral education plays a vital role in cultivating the world’s future innovators, yet doctoral students face elevated risks of psychological distress (Evans et al., 2018). Although existing research has documented the prevalence of such challenges, it remains largely confined to a pathological paradigm that conceptualizes psychological resilience as a static trait. Rooted in a deficit model, this approach relies primarily on cross-sectional quantitative data to identify risk factors and symptoms, thereby isolating resilience from the situated academic contexts in which it is dynamically developed and sustained. Consequently, the core mechanisms through which individuals adapt and grow through adversity remain inadequately explained. While scholars have increasingly called for process-oriented and ecological perspectives (Bergeman et al., 2021), a coherent framework that elucidates how internal psychological dynamics and external ecosystems interact to foster resilience in high-pressure doctoral environments remains lacking (Xu et al., 2024).

To address this gap, this study adopts a positive psychology and ecological systems perspective. We aim to examine how doctoral students who transition from “surviving” to “thriving” construct psychological resilience within demanding academic settings. Our central research question is: How is psychological resilience progressively developed in such environments, and what key stages, core driving mechanisms, and ecosystem interactions characterize this process? By addressing this question, this study seeks to advance the theoretical orientation of resilience research from a trait-based description toward a process-mechanism explanation.

## 2 Literature review and theoretical framework

### 2.1 The conceptual evolution of psychological resilience: from trait to process to system

Research on psychological resilience has evolved through three broad conceptual phases (Hill et al., 2025). First, early trait-based approaches viewed resilience as a stable personality attribute (Silveira et al., 2022), focusing primarily on identifying intrinsic protective factors—such as optimism and self-confidence**—**that characterize resilient individuals. While this perspective helped catalog positive psychological qualities, its deterministic assumptions and limited attention to contextual complexity restricted its ability to explain variations in resilience across situations and over time.

A second paradigm emerged with the advent of process-oriented theories, which reconceptualized resilience not as a fixed trait, but as a dynamic process of adaptation through ongoing interaction with adversity (Ungar and Theron, 2020). This perspective reframed resilience as a developmental trajectory involving active meaning-making and agency. However, although process models highlighted temporal dynamics, they often underemphasized the role of multi-level ecosystems and the specific mechanisms through which contextual factors shape resilient outcomes.

The most recent paradigm adopts a systems perspective, situating resilience within broader ecological frameworks (Ventriglio et al., 2025). Drawing on ecological systems theory (Maltby, 2025), resilience is understood as an emergent property of the complex, reciprocal interactions between individuals and their environments. Ungar’s (2015) social-ecological model further emphasizes that resilience depends not only on individual qualities but also on the accessibility and meaningfulness of environmental resources. Yet, while existing ecological models usefully describe structural and resource conditions, they offer limited insight into the proximal processes through which individuals engage with these resources to foster resilience over time.

This systems view—with its emphasis on person–context interactions**—**naturally aligns with theories of identity formation. Adaptive functioning in challenging environments invariably involves processes of role internalization and value negotiation. In academic contexts, the development of psychological resilience is thus deeply intertwined with the formation of a scholarly identity. Grounded in self-determination theory (Li et al., 2025), resilience is facilitated when learning environments support individuals’ basic psychological needs for autonomy, competence, and relatedness, thereby fostering motivation, identity integration, and well-being. Synthesizing these perspectives, we conceptualize doctoral resilience as the capacity to adapt and grow amid academic adversity through the dynamic integration of personal values, social resources, and an evolving academic identity.

### 2.2 Academic ecology and psychological challenges of doctoral students

#### 2.2.1 International challenges in doctoral psychological adaptation

Doctoral students operate within an academic environment characterized by structural tensions and high psychological demands, forming a distinct context for the development of resilience. These challenges are internationally prevalent, consistently documented in studies across multiple national contexts (Mackie and Bates, 2019; Hazell et al., 2020; Cherry et al., 2024).

First, a fundamental tension exists between the inherent uncertainty of research tasks and highly structured evaluation systems. Doctoral training centers on original knowledge production, which is intrinsically uncertain and exploratory. Yet, academic assessment often relies on short-term, quantifiable metrics, creating a paradox between open-ended inquiry and standardized measurement that serves as a core academic stressor (Woolston, 2022).

Second, the culture of hyper-competition for limited resources and positions carries the risk of eroding the sense of academic meaning. When instrumental rationality—exemplified by the excessive pursuit of publication counts and impact factors—overshadows value rationality rooted in intellectual curiosity, the intrinsic significance of academic work can diminish, leading to widespread burnout (Mackie and Bates, 2019; Slimi et al., 2024).

Third, doctoral students face compound pressures stemming from multiple social roles and complex support relationships (Mills et al., 2024). They must navigate conflicting roles as learners, researchers, and project managers, while the advisor-advisee relationship—a central support mechanism—operates within an asymmetric power structure. While a positive mentoring relationship can offer crucial cultural capital and emotional support, a dysfunctional one often becomes a primary source of psychological strain (Friedrich et al., 2023; Wollast et al., 2023; Guo, 2025).

#### 2.2.2 Research gaps and the focus of this study

Despite these distinctive ecological pressures, research on doctoral students’ psychological resilience remains limited in several key respects. Conceptually, studies often adopt a de-contextualized approach, relying on generic scales that fail to capture resilience as a situated process of continual meaning-making and strategic action within high-pressure academic environments. Theoretically, a negative bias persists, with most work framed within a pathological “defense–recovery” paradigm, thereby overlooking the mechanisms that enable certain subgroups to achieve psychological flourishing. Although Ungar’s (2015) social-ecological model and Masten’s (2001) process model offer valuable macro perspectives—conceptualizing resilience as ordinary, dynamic person–context transactions—a coherent framework that integrates process and ecology to elucidate the micro-level interactions between doctoral students’ internal drives and their external environments remains underdeveloped. It is this critical gap in understanding the mechanisms of person–context interaction that constitutes the central focus of the present study, which seeks to extend existing ecological and process models by specifying micro-level interaction patterns within the doctoral context.

## 3 Research design

This study employed constructivist grounded theory (Charmaz, 2006, 2014; Kuek et al., 2024) as its core methodology, following its ontological premise of multiple realities co-constructed through researcher–participant interaction. This approach is grounded in the philosophical position that psychological resilience is not a pre-existing static trait, but a co-constructed system of meaning and behavioral patterns that emerges from continuous interaction between individuals and their academic environments. Theoretical categories were not pre-existing in the data but were actively interpreted and negotiated during iterative interviewing and coding. This perspective allows for an in-depth exploration of how individuals interpret their experiences and how these interpretations shape their actions, making it highly congruent with our aim to investigate complex dynamic processes and develop a substantive theoretical model.

The study was conducted with doctoral students from three high-level universities in China. The high-pressure academic setting in which these students are embedded provided a fertile context for observing the dynamic construction of psychological resilience.

Researcher positionality and reflexivity are critical in constructivist research (Charmaz, 2006, 2014). All members of the research team possess social science backgrounds and have personal experience with graduate study. While this shared background facilitated a deeper understanding of the doctoral experience, it also carried the risk of presupposing what constitutes “successful adaptation.” To manage this positionality and ensure that the theory was grounded in the data, we implemented several systematic strategies: (1) documenting initial assumptions at the study’s outset; (2) holding regular team discussions during coding to actively search for disconfirming evidence; and (3) maintaining reflective memos to track analytical decisions. For instance, our initial coding framework, influenced by the deficit-oriented literature dominating doctoral education research, tended to foreground individual vulnerabilities when interpreting advisor-related stress. Through systematic memoing and team discussions, we actively challenged this lens, which enabled the balanced identification of both “relational inhibition” and “relational compensation” patterns.

### 3.1 Participants and sampling strategy

Guided by the principles of constructivist grounded theory, we employed an iterative sampling strategy combining purposive and theoretical sampling (Charmaz, 2006; Stratton, 2024). The initial purposive sampling targeted doctoral students who met the following criteria: (a) being recommended by supervisors or peers as exhibiting a positive research mindset or skill in navigating adversity; (b) self-reporting significant research setbacks that they had subsequently overcome or effectively managed; and (c) presenting a current state of “successful adaptation.”

“Successful adaptation” was operationally defined along two dimensions: (a) Objective Progress: Demonstrable academic advancement following a setback, such as a manuscript acceptance, an experimental breakthrough, or the completion of a key research milestone. (b) Subjective Narrative Patterns: Interview narratives exhibiting a sense of agency, reflective depth, and a predominantly positive emotional tone. This was assessed by examining the use of proactive coping narratives, the ability to articulate lessons learned from challenges, the presence of stable and positive affect, and the description of specific, effective coping strategies.

As data analysis progressed, theoretical sampling was employed to deliberately recruit participants who could help develop and refine emerging conceptual categories (e.g., “value transcendence”). This iterative process continued until theoretical saturation was achieved, indicated by (a) no new properties or dimensions emerging from core categories, (b) relationships between categories becoming well-established, and (c) further data collection seeming redundant (Charmaz, 2006). The final sample size stabilized at 24 participants (see Table 1). To verify saturation, we independently analyzed four withheld interviews, which confirmed that the core categories were robust, well-developed, and stable.

**Table 1:** Demographic and academic characteristics of participants (*N* = 24).

Variable	Category	n	%
Gender	Male	11	45.8
	Female	13	54.2
Year of study	First year	4	16.7
	Second year	6	25.0
	Third year	8	33.3
	Fourth year and above	6	25.0
Academic discipline	STEM	14	58.3
	Humanities and social sciences	10	41.7
Institution type	Comprehensive University	8	33.3
	STEM-Oriented University	9	37.5
	Humanities and Social Sciences University	7	29.2
Supervisory style	Close supervision	8	33.3
	Autonomous exploration	10	41.7
	Task-oriented	6	25.0

### 3.2 Data collection

Data were primarily collected through semi-structured in-depth interviews (Balzen et al., 2025), supplemented by analysis of textual materials participants provided, including reflective research journals (*n* = 12) and personal notes on research setbacks (*n* = 8) to enable data triangulation. The textual materials were analyzed using the same constant comparative method and coding framework applied to interview transcripts, serving to corroborate, contextualize, and sometimes challenge the interview narratives. Triangulation served to contextualize retrospective accounts within real-time experience. For example, Participant B07’s research journal documented immediate emotional turmoil following a failed experiment, providing a real-time account that contrasted with his retrospective interview narrative of “calmly analyzing the failure.” This comparison revealed cognitive restructuring as an unfolding process, rather than a neat retrospective summary. This comparison thus served both a methodological function—contextualizing retrospective accounts—and an analytic function—revealing cognitive restructuring as an unfolding process (Charmaz and Thornberg, 2021).

This study has been approved by the Ethics Review Committee of Anhui Normal University. Prior to interviews, all participants provided written informed consent. Throughout the research process, we strictly maintained confidentiality and implemented thorough data anonymization procedures. Any identifiable information was anonymized and replaced with coded identifiers (e.g., Student B01, B02, …).

Conducted in a secure, confidential, and empathetic setting, interviews averaged 60 min in duration. Employing a narrative interview approach (Harness et al., 2025), we guided participants to reconstruct critical event sequences of “setback-adaptation-growth” from their doctoral journeys. The core interview protocol included: “Could you describe a pivotal event in your doctoral research that caused you significant stress or feeling stuck?” “Facing this situation, how did you think, feel, and act? What key individuals or resources influenced you?” and “Reflecting on this experience, how do you understand its significance in your doctoral development and broader life trajectory?” All interviews were audio-recorded and verbatim transcribed, resulting in a textual corpus of approximately 160,000 words.

### 3.3 Data analysis

Data analysis followed an iterative, cyclical process that engaged continuously with data collection, adhering to the three-stage coding process of constructivist grounded theory, comprising initial, focused, and selective coding. The entire analytical process was managed using NVivo 12 software (Wang and Guo, 2024).

#### 3.3.1 Initial coding

We conducted line-by-line analysis of all interview transcripts, initially generating 402 reference nodes through labeling and conceptualization. Using the constant comparative method, we consistently interrogated phenomena emerging from the data and compared experiences across participants. Through merging and clustering these nodes, we distilled 26 initial concepts, which were further synthesized into 13 preliminary categories (Table 2).

**Table 2:** Initial coding: initial codes and preliminary categories.

Preliminary category	Initial code	Representative quotations	Reference points
Task pressure	Research task overload	“I have to complete multiple tasks every week-literature review, experiments, data analysis, thesis writing. I constantly feel chased by deadlines.”	22
	Time resource strain	“Working over 12 h daily, including weekends, with no personal life. My body and mind are in a state of constant depletion.”	20
Competitive pressure	Outcome comparison anxiety	“My peers have already published several top-journal papers, while I’m still revising my first one. This gap makes me extremely anxious.”	18
	Resource scarcity pressure	“We have to compete for lab equipment, supervisor time, and project slots. This internal competition is exhausting.”	16
Identity confusion	Self-efficacy doubt	“I often feel inadequate when tackling complex research problems and question if I have the academic capability required for a PhD.”	19
	Role identity ambiguity	“I struggle to switch between multiple roles-researcher, student, project lead-and cannot find a clear sense of self.”	17
Career anxiety	Unclear career prospects	“Academic positions are shrinking, and industry requirements are vague. I’m deeply uncertain about my future career path.”	16
	Cost–benefit anxiety	“I’ve invested five prime years in my PhD. Seeing my peers already successful in their careers makes me question my choice.”	14
Metacognitive development	Thought process monitoring	“I began consciously noticing my negative thought patterns and learned to actively interrupt this self-critical internal dialogue.”	18
	Cognitive strategy adjustment	“When stuck, I can detach from the immediate problem and seek solutions from a broader perspective.”	16
Meaning transformation	Adversity reappraisal	“Although repeated experimental failures delayed progress, they deepened my understanding of the research question-a more fundamental gain.”	17
	Challenge reframing	“I now view strict academic critiques as opportunities to improve research quality, not as personal failure.”	15
Value establishment	Internal standard formation	“I gradually built my own evaluation system centered on knowledge contribution and innovation, rather than blindly chasing publication numbers.”	14
	Research purpose clarification	“I reconnected with the curiosity and passion that initially drew me to research, rediscovering the intrinsic motivation for my work.”	12
Goal management	Task decomposition	“I break down the monumental dissertation into manageable, evaluable sub-tasks, which reduces the psychological barrier to execution.”	19
	Time management optimization	“I use time-blocking to allocate specific periods for different research tasks, which significantly improves efficiency.”	17
Emotion regulation	Stress alleviation techniques	“I manage anxiety through mindfulness meditation and regular exercise to maintain psychological balance.”	16
	Emotional acceptance	“I acknowledge and accept negative emotions during research, learning to coexist with them rather than fight them.”	14
Support seeking	Advisor communication	“I proactively update my supervisor on progress and difficulties, seeking both professional guidance and emotional support.”	17
	Peer support networks	“We established regular communication channels with lab mates for mutual academic and emotional support.”	15
Purpose realization	Research value identification	“Realizing my research could provide insights for solving real-world problems gives deeper meaning to my daily efforts.”	13
	Societal contribution awareness	“Connecting my personal research to broader societal needs helps me understand the value of academic work on a larger scale.”	11
Resilience internalization	Resilience internalization	“After repeated setbacks, I’ve developed the psychological capacity to learn from failure and recover quickly.”	14
	Growth mindset cultivation	“I now perceive challenges as opportunities to enhance my abilities and believe I can overcome them through effort.”	12
Prosocial contribution	Experience sharing	“I actively share research experiences and lessons with junior graduate students to help them adapt to academic life.”	11
	Academic community building	“I engage in building our lab culture to create a mutually supportive, collectively growing academic community.”	9

#### 3.3.2 Focused coding

Through constant comparison and iterative questioning of the data, we refined the most significant and analytically promising initial codes into more abstract focused codes. This process involved assessing which codes best captured recurring patterns across participants’ experiences and elevating them to conceptual categories. Through this iterative process, a coherent narrative structure emerged: the high-pressure academic environment combined with setback events triggered doctoral students’ stress perception. In response, individuals engaged in cognitive restructuring and strategy integration, ultimately leading toward value transcendence. This analytical phase yielded four main categories and their interrelationships (see Table 3), establishing the foundation for identifying the core category in the subsequent selective coding process.

**Table 3:** Results of focused coding: main categories and core connotations.

Main category	Corresponding stage	Subcategories (reference points)	Core connotation
Stress Perception	Stage 1	Task pressure (42); competitive pressure (34); identity confusion (36); career anxiety (30)	Systemic imbalance and crisis triggering: The perception of a significant gap between research demands and personal resources/capabilities serves as the initial condition for resilience construction.
Cognitive Restructuring	Stage 2	Metacognitive development (34); meaning transformation (32); value establishment (26)	Transformation of meaning systems and identity negotiation: The active reconstruction of the meaning of adversity and academic work, facilitating the identity shift from “student” to “researcher.”
Strategy Integration	Stage 3	Goal management (36); emotion regulation (30); support seeking (32)	Systematic agency demonstration: The integration of internal and external resources through concrete behavioral strategies to cope with stress, thereby validating and reinforcing new cognitive frameworks.
Value Transcendence	Stage 4	Purpose realization (24); resilience internalization (26); prosocial contribution (20)	Life meaning expansion and academic identity formation: Connecting personal academic work to broader values, where resilience becomes an internalized capacity manifested through prosocial behaviors.

Reference points indicate the frequency with which each initial category was referenced in the interview transcripts.

#### 3.3.3 Selective coding

Through systematic analysis of all categories and their interrelations, “process-ecological dynamic construction” emerged naturally as the core category capable of integrating all phenomena. This category proved most potent in capturing the essence of our data, as it organically incorporated both the temporal sequence of resilience development (four-stage process) and its contextual embeddedness (ecosystem interactions). Around this core category, we developed the final theoretical storyline: Doctoral students in high-pressure academic environments, driven by the dual engines of “meaning-making” and “agency activation,” navigate a nonlinear, four-stage process deeply embedded within dynamic interactions across individual, relational, and institutional ecosystems, while co-evolving with their academic identity formation.

To ensure research rigor, coding was performed independently by two researchers trained in qualitative methods. Discrepancies were resolved through discussion until consensus was achieved. Inter-coder reliability was assessed by calculating the percentage agreement between the two independent coders. The obtained coefficient exceeded commonly accepted thresholds, indicating acceptable reliability (Miles and Huberman, 1994; Hemmler et al., 2022).

## 4 Research findings: a process–ecological model of psychological resilience

**Figure 1:**
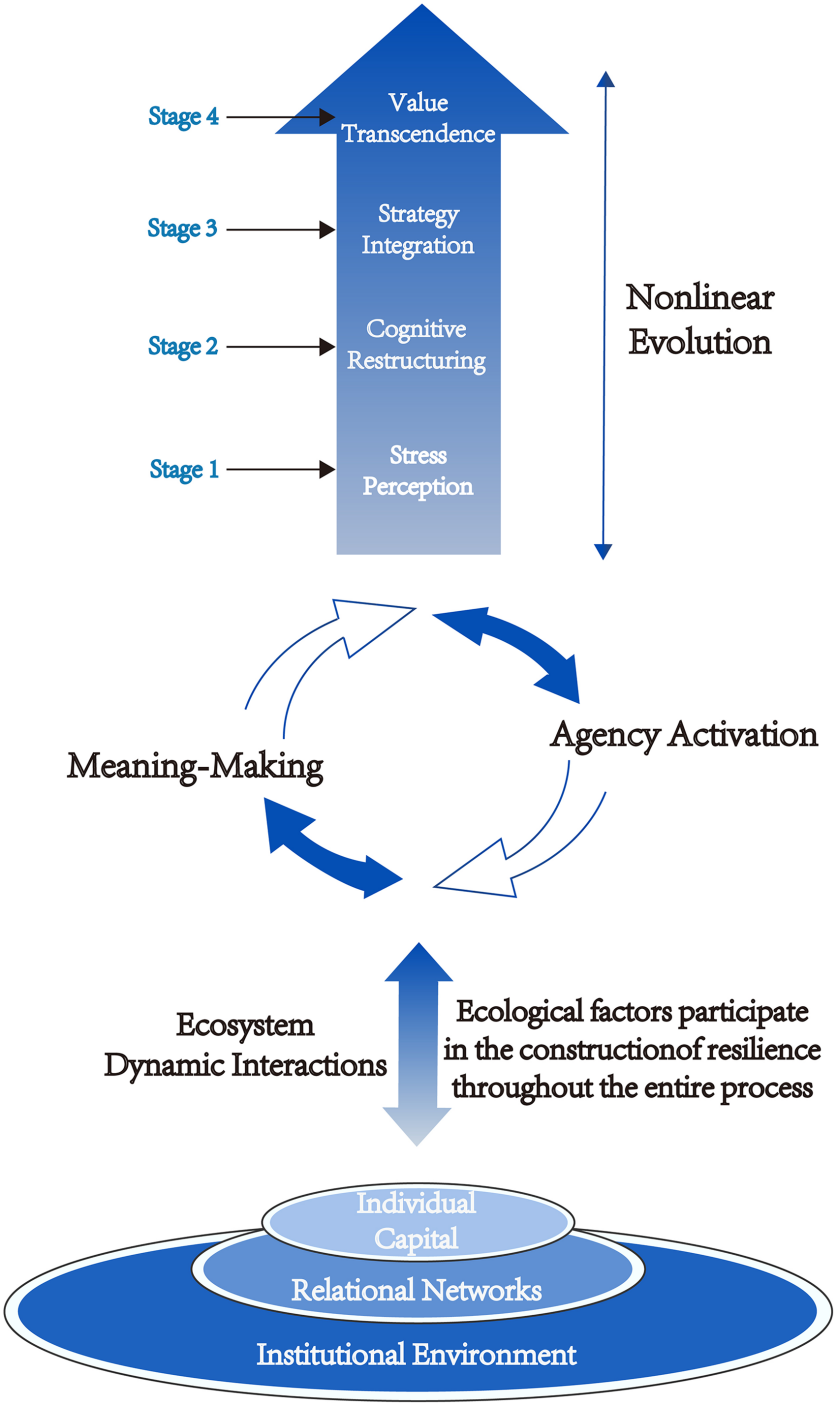
A process-ecological model of developing psychological resilience in doctoral students.

Through systematic analysis of interview data, we developed a process-ecological model of doctoral students’ psychological resilience (Figure 1). The model reveals that resilience is not a static trait but rather a developmental trajectory that emerges through continuous individual-ecosystem interactions within high-pressure academic environments. Characterized by nonlinear progression, this trajectory reflects movement from a reactive state of “surviving” under pressure toward a proactive state of “thriving.”

### 4.1 From surviving to thriving: the four-stage nonlinear development

It is crucial to emphasize that progression through these four stages is inherently nonlinear, dynamic, and iterative. Participants’ experiences revealed how individuals might cycle between stages when transitioning between research tasks (e.g., B04), skip stages with robust external support (e.g., B11), or even regress temporarily following significant new setbacks. This pattern confirms that resilience development is not a linear achievement but an ongoing practice navigated through adversity.

#### 4.1.1 Illustrative case—cycling between stages

Participant B04, after completing a paper, could clearly articulate the social value of their research (indicative of the value transcendence stage). However, when beginning a new project involving unfamiliar methodologies, they experienced renewed self-doubt (reverting to the stress perception stage). By consciously recalling past successes and proactively consulting methodological experts, they engaged in strategy integration, thereby restoring confidence and demonstrating dynamic equilibrium between stages.

#### 4.1.2 Illustrative case—stage skipping

Following paper rejection (a stress perception event), Participant B11 received immediate, substantial support from their supervisor. This effective mentoring prevented deep self-criticism and enabled direct progression into strategy integration (seeking help, revising the manuscript), effectively bypassing the intensive independent cognitive restructuring typically characterizing that transition.

#### 4.1.3 Illustrative case—accelerated recovery

Participant B06, after publishing and reflecting on her work’s societal value (value transcendence), faced repeated experimental failures and reverted to self-doubt (stress perception). Crucially, this regression was shorter-lived than initial setbacks, stating: “The failure reactivated old insecurities, but I bounced back faster.” This illustrates how resilience accumulates through experience, making subsequent regressions less debilitating.

The following sections detail each stage’s core categories and characteristics, supported by representative excerpts from the data.

#### 4.1.4 Stage 1: stress perception—system imbalance and crisis trigger

Doctoral students navigate multiple pressures arising from academic tasks, competitive environments, identity formation, and career uncertainty, leading to psychological system imbalance. This stage centers on the subjective appraisal of the discrepancy between academic demands and personal resources. This perception—both a reflection of objective conditions and subjective evaluation—constitutes the initial condition for resilience development, revealing the identity tension between their roles as “learners” and “researchers.” Task pressure manifests as overload and chronic time scarcity, exemplified by B09: “Juggling literature review, experiments, data analysis, and writing every week, constantly racing against deadlines leaves me perpetually drained, both mentally and physically.” Competitive pressure emerges through social comparison and resource competition, as B17 noted: “While my peers have published three top-tier papers, I’m still revising my first manuscript… even lab equipment and my supervisor’s time require negotiation.” Identity confusion and career anxiety further compound psychological strain, with B14 observing: “Complex research problems trigger self-doubt. With scarce academic positions and ambiguous industry requirements, I feel profound anxiety about my future.”

#### 4.1.5 Stage 2: cognitive restructuring—meaning system transformation and identity negotiation

When external coping strategies prove insufficient, resilience development shifts inward toward profound cognitive restructuring. This stage represents an intensive process of academic identity negotiation. Metacognitive development—the monitoring and adjustment of thought patterns—signals the awakening of agency, as reflected in B05’s account: “I consciously notice and interrupt the ‘I’m not capable’ narrative, challenging its validity by asking, ‘What evidence supports this belief?’ This helps me reclaim cognitive control.” Meaning transformation involves fundamentally re-evaluating the value of academic work, as demonstrated by B12 reframing failure as “essential learning.” This represents an identity reorientation from valuing “output success” to prizing “exploratory process”: “Although experimental failures delayed progress, they deepened my understanding. Redefining them as necessary learning fundamentally transformed my research approach.” Value establishment creates an internal academic compass, marking the transition from passive student to proactive “scholar-in-training,” evidenced by B21’s statement: “I’ve developed an evaluation system centered on knowledge contribution and innovation—no longer chasing publication counts but reconnecting with scientific curiosity.” This cognitive restructuring provides the psychological foundation for subsequent action.

#### 4.1.6 Stage 3: strategy integration—agency actualization and practice-based identity formation

Building upon the cognitive foundation established previously, doctoral students systematically actualize their agency by translating internal reframing into external action. This stage proves crucial for forming an identity-in-practice. Goal management involves converting abstract threats into manageable steps. As B08 described: “Deconstructing my dissertation into actionable sub-tasks and using time-blocking methods significantly enhanced both efficiency and perceived control.” Emotion regulation involves managing distress, with B16 sharing: “Through mindfulness and regular exercise, I’ve learned to accept—rather than resist—negative emotions, coexisting with them.” Support seeking carries deeper social significance, representing intentional identity transition from “isolated striver” to “community member.” B11 emphasized: “I proactively update my supervisor about progress and challenges, and maintain regular communication with lab colleagues.” Successful strategy implementation not only addresses immediate problems but also validates and reinforces the emerging identity and cognitive frameworks developed in Stage 2.

#### 4.1.7 Stage 4: value transcendence—meaning expansion and academic identity formation

The critical shift from Stage 3 to Stage 4 lies in the object of agency—from task mastery (“How do I fix this paper?”) to identity enactment (“What kind of scholar am I becoming?”). This stage marks the maturation of resilience into an enduring orientation toward flourishing, accompanied by consolidated academic identity—representing the qualitative transition from surviving to thriving. Purpose realization connects personal research to broader societal values, reflecting identity crystallization. B19 articulated: “Recognizing that my algorithmic research could optimize healthcare resource allocation infused mundane programming with profound meaning.” Resilience internalization indicates that adaptive capacity has become stable psychological capital, with B06 noting: “Repeated setbacks forged my ability to learn from failure and recover quickly**—**this resilience matters more than any single publication.” Prosocial contribution manifests through altruistic behaviors, demonstrating how stabilized resilience reciprocally enriches the ecosystem. B23 expressed: “Mentoring junior graduate students deepens my self-understanding while helping others, completing my transition from beneficiary to benefactor.”

### 4.2 Core mechanisms: the synergy of multiple dynamics

#### 4.2.1 The dual-engine drive: meaning-making and agency activation

The development of psychological resilience is propelled by a synergistic positive feedback system comprising “meaning-making” and “agency activation” that operates across all stages. This dual-engine mechanism specifies how meaning-making establishes the value orientation and cognitive foundation for resilience, while agency activation supplies the behavioral impetus and strategic execution. Together, they form the core psychological system enabling individuals to navigate adversity.

First, meaning-making demonstrates hierarchical progression. Its content evolves from initial instrumental purposes focused on “survival” (e.g., “persisting to obtain the degree”) toward valued “flourishing” purposes (e.g., “contributing new knowledge to the field” or “serving society through research”). This elevation in meaning provides more enduring and autonomous psychological energy, directly shaping the direction and quality of agentic practices. As Participant B18 reflected, illustrating this shift in meaning orientation: “When I viewed my doctoral journey merely as a ‘degree-obtaining process’ (instrumental meaning), every setback felt unbearable, and my actions were passive and resistant. But after redefining it as ‘forging a scholar’s mindset and research character’ (valued meaning), this new perspective enabled me to proactively investigate the principles behind failures.”

Second, agency activation exhibits developmental staging. It progresses from reactive responses under pressure (e.g., compulsory task completion) to proactive adaptation following cognitive restructuring (e.g., goal management, emotion regulation), culminating in generative actions during the value transcendence stage (e.g., actively building support networks, engaging in community development). Each successful agentic practice, in turn, verifies, strengthens, and elevates the underlying meaning system. As B18 further explained: “When my proactive experimentation with new methods finally yielded a breakthrough (successful agency), my conviction in my chosen path and research values was powerfully reinforced (meaning system strengthening). This renewed belief then motivated me to confront subsequent challenges with greater initiative.”

Thus, this dual engine constitutes a self-reinforcing cycle: deeper meaning fuels more proactive agency, while successful agentic practices consolidate more profound meaning. It is precisely this iterative process that drives progressive development across the stages.

#### 4.2.2 Ecological foundations: the triadic interaction of individual capital, relational networks, and institutional environment

The construction of psychological resilience is deeply embedded within a multi-level ecosystem. Our analysis reveals dynamic cross-level interactions among individual capital (e.g., academic skills, optimism), relational networks (e.g., mentors, peers, family), and the institutional environment (e.g., evaluation systems, program structures, cultural climate). From interview data, we identified three prototypical interaction patterns illustrating these dynamics.

#### 4.2.3 Synergistic reinforcement pattern

When individual capital, relational networks, and institutional environment all function optimally, they create mutually reinforcing conditions that establish the most favorable pathway for resilience development. As B13 described: “When my theoretical framework had flaws, my supervisor’s guidance precisely addressed these conceptual limitations (relational network). The faculty’s academic exchange fund provided necessary resources (institutional environment), while my prior preparation enabled me to quickly integrate this feedback (individual capital) —together creating a comprehensive support system.”

#### 4.2.4 Compensatory pattern

When support is deficient at one system level, strengths at other levels can effectively buffer these limitations. This demonstrates the resilience system’s plasticity and resource substitutability. B07’s experience illustrates this functional compensation: “The university’s mid-term evaluation criteria were exceptionally rigorous (institutional challenge), but my supervisor provided substantial trust and intellectual freedom (relational compensation). Simultaneously, I maintained psychological equilibrium through consistent exercise (individual compensation), enabling me to navigate this period successfully.”

#### 4.2.5 Inhibitory pattern

Serious deficiencies at one system level—particularly in relational networks—can suppress or even nullify resources available at other levels. B20’s situation exemplifies this “weakest link” effect: “Despite my extensive literature knowledge (individual capital) and the college’s open seminar policy (institutional environment), my supervisor’s persistent criticism and poor communication (relational inhibition) prevented me from effectively utilizing these resources, substantially eroding my confidence.” This pattern reveals how the most deficient system level may determine the upper threshold for resilience development—an important qualification to ecological theories.

#### 4.2.6 Environmental optimization: the psychological ecology of challenge-support balance

Given that ecosystem constraints—particularly at the relational level—can constrain resilience, the question becomes how to optimize these interactions. The efficacy of this triadic ecosystem ultimately manifests through the dynamic balance between environmental challenges and supports. Effective resilience cultivation depends on this balance creating an optimal “zone of psychological tension,” where moderate challenges activate growth needs while appropriate, timely supports provide necessary resources and security.

First, the challenge-support ratio directly shapes resilience development trajectories. Excessive challenge with inadequate support—as seen in B20’s inhibitory pattern—often traps individuals at the stress perception stage. Conversely, excessive support with insufficient challenge fails to stimulate the cognitive restructuring and strategy integration essential for resilience maturation.

Second, achieving balance requires enabling support rather than protective insulation. As B15 articulated, the ideal condition emerges when “supervisors set high-challenge goals while granting substantial autonomy and providing timely support.” Here, high challenges create the imperative for cognitive restructuring; substantial autonomy directly activates student agency; and timely support ensures psychological safety. These three elements collectively drive the meaning-making/agency activation dual engine.

Finally, this principle must be systematically embedded across the individual-relational-institutional ecosystem. Relationally, this means combining high expectations with high responsiveness. Institutionally, it requires pairing rigorous academic standards with systematically available support resources, thereby creating a supportive high-pressure environment.

Consequently, skillful navigation of the challenge-support dynamic represents both the practical core and crucial leverage point for transitioning from remedial interventions toward developmental ecosystem construction.

## 5 Discussion

### 5.1 Theoretical contributions

#### 5.1.1 Advancing resilience theory: conceptualizing resilience as identity-driven meaning-making

The process-ecological model developed in this study refines and operationalizes existing resilience theories (Masten, 2001; Ungar, 2015) within the underexamined context of doctoral education. Its primary contribution lies in revealing how, within high-pressure academic contexts, resilience development and academic identity formation represent deeply intertwined processes. Each cognitive adjustment and strategic action in response to adversity simultaneously constitutes a practical response to the fundamental identity question: “What kind of researcher am I becoming?” This conceptualization extends these identity theories by framing resilience as identity investment (London, 1983)—the process whereby individuals persist because the academic role has become integral to their self-concept, beyond the possession of coping skills. Consequently, the transition from surviving to thriving represents an identity transformation—from a passive student enduring pressure to an active scholar generating knowledge and contributing to their community.

By explicitly framing the stages of “cognitive restructuring” and “value transcendence” as processes of identity negotiation and consolidation, our model provides a more profound explanatory framework for understanding resilience: as the ongoing practice through which individuals defend, refine, and realize their emergent ideal professional identity amidst adversity.

#### 5.1.2 Innovating doctoral education research: a process-ecological lens on micro-mechanisms

Our model extends, rather than replaces, existing frameworks by integrating process and ecological perspectives within the specific context of doctoral education. It operationalizes identity work into observable micro-mechanisms, thereby bridging the theoretical divide between process-oriented and ecological perspectives on resilience. First, the dual-engine system of meaning-making and agency activation substantially enriches existing process models. It delineates a positive feedback cycle wherein the elevation of meaning fuels more proactive agency, whose successes in turn validate and deepen the meaning system. This explains how internal motivational states act as crucial mediators, translating external ecological resources into sustained developmental momentum, thereby contextualizing and extending social cognitive theory within academic resilience contexts (Bandura, 1986). While this dual engine may reflect a potentially universal resilience process, its specific manifestations are likely shaped by local ecological conditions—a boundary condition we examine in the limitations.

Simultaneously, the identified triadic interaction patterns—synergistic reinforcement, compensation, and inhibition—provide processual, micro-level evidence for ecological systems theory. They demonstrate that doctoral resilience constitutes an emergent property of complex, nonlinear system interactions. The inhibitory pattern, in particular, reveals the “weakest-link effect” within ecosystems, suggesting that deficiencies in one system level (especially relational networks) can critically constrain resilience development regardless of strengths elsewhere. This insight underscores the necessity of adopting a whole-ecosystem intervention approach, as initiatives targeting single levels may yield limited returns.

Furthermore, by grounding the model explicitly within the high-pressure, high-cognitive-demand context of doctoral education, we avoid the vagueness of overly generic models and achieve the contextual specificity essential for robust theoretical development, thereby establishing meaningful connections with theories of academic socialization and workplace learning (Weidman et al., 2001; Austin, 2002).

### 5.2 Practical implications and policy recommendations

#### 5.2.1 Empowering individuals: catalyzing the dual-engine system

Institutional support systems should actively catalyze both meaning-making and agency activation. This necessitates moving beyond conventional skills training to establish workshops focused on metacognitive development and academic narrative restructuring, guiding doctoral students in clarifying academic values and reframing adversities. Concurrently, systematic instruction in instrumental coping strategies—such as goal management and emotion regulation—should provide actionable methods for exercising agency. These interventions directly target the dual-engine mechanism (meaning-making and agency activation) presented in “Section 4.2.1.”

#### 5.2.2 Activating relationships: cultivating supportive academic communities

Universities should deliberately institutionalize supportive academic relationships. Central to this endeavor is reforming supervisory practices by integrating developmental mentoring and competencies in maintaining challenge-support balance into advisor training and evaluation criteria. Furthermore, establishing structured peer-support systems—such as “research incubator groups” and “peer support networks” facilitated by senior doctoral students—can effectively formalize the transmission of tacit knowledge and the provision of emotional support. Such initiatives operationalize the relational network component of our triadic ecosystem (Section 4.2.2).

#### 5.2.3 Optimizing systems: fostering a resilience-oriented educational ecology

Systemic reform should comprehensively embed resilience development into the educational ecosystem. Key priorities include transforming evaluation systems by reducing reliance on high-stakes summative assessments and enhancing formative feedback mechanisms. Academic processes should be redesigned to pair high-challenge milestones (e.g., qualifying examinations) with readily accessible, institutionalized support resources (e.g., writing centers, methodological consulting). Ultimately, by publicly recognizing exemplars of resilience, collaboration, and altruism, institutions can reshape pluralistic definitions of success and cultivate a more constructive academic culture. This recommendation addresses the inhibitory pattern (Section 4.2.2) and embodies the challenge-support balance principle (Section 4.2.3).

## 6 Research limitations and future directions

While providing valuable insights, this study has several limitations that suggest productive avenues for future research. First, our sampling strategy focused on individuals demonstrating “successful adaptation,” potentially introducing survivorship bias (Elston, 2021). Consequently, our model may more clearly delineate protective pathways while underrepresenting critical risk mechanisms and tipping points leading to psychological exhaustion or attrition. Specifically, our sample likely obscures critical junctures—such as the point where “strategy integration” fails and learned helplessness consolidates, or the persistent identity dissonance that leads to attrition without ever reaching the “value transcendence” stage. Future research employing extreme-case comparison designs—contrasting “thrivers,” “persistent strugglers,” and “dropouts”—could offer more dialectical understanding of resilience development.

Second, the retrospective interview methodology, while valuable, cannot completely circumvent potential post-hoc rationalization in participant narratives. Intensive longitudinal designs with repeated measures at critical doctoral milestones would more precisely capture dynamic resilience trajectories and transitional tipping points. Additionally, the cross-sectional retrospective design cannot establish temporal precedence between cognitive restructuring and agency activation; experience sampling methods (ESM) are needed to test reciprocal causality (Larson and Csikszentmihalyi, 2014).

Finally, while the cultural embeddedness of our model represents a distinctive strength, it also delineates its boundaries. The dual engine of meaning-making and agency activation reflects a potentially universal resilience process, but its specific manifestations—such as the pronounced role of family support and heightened sensitivity to hierarchical mentoring—may be culturally accentuated. Cross-cultural validation and comparative designs in individualistic academic contexts are needed to distinguish universal mechanisms from culturally specific pathways.

## 7 Conclusion

The process-ecological model developed in this study fundamentally reconceptualizes doctoral resilience from a static trait to a dynamic socio-cultural practice that co-evolves with academic identity within high-pressure environments. This practice follows a nonlinear developmental sequence—stress perception, cognitive restructuring, strategy integration, and value transcendence—propelled by the dual engine of meaning-making and agency activation, all deeply embedded within dynamic interactions across individual, relational, and institutional ecosystems.

This theoretical reconceptualization necessitates a crucial paradigm shift in doctoral student support: from individually-targeted remedial “fixes” toward systemically cultivating empowering ecosystems; from fragmented interventions toward deliberately engineered environments maintaining optimal challenge-support balance. Beyond providing novel theoretical perspectives and practical roadmaps for addressing the doctoral mental health crisis, our integrated process-ecological framework offers a transferable analytical lens for understanding professional development in other high-stakes, high-investment fields including medicine, law, and scientific research.

## Data Availability

The original contributions presented in the study are included in the article/supplementary material, further inquiries can be directed to the corresponding author.
